# Computational Identification of Essential Enzymes as Potential Drug Targets in *Shigella flexneri* Pathogenesis Using Metabolic Pathway Analysis and Epitope Mapping

**DOI:** 10.4014/jmb.2007.07006

**Published:** 2020-12-14

**Authors:** Priyanka Narad, Himanshu, Hina Bansal

**Affiliations:** Amity Institute of Biotechnology, Amity University Uttar Pradesh, Sector 125, Noida-201303, U.P., India

**Keywords:** Essential enzymes, in silico comparative analysis, KEGG database, metabolic pathway analysis, subcellular localization

## Abstract

*Shigella flexneri* is a facultative intracellular pathogen that causes bacillary dysentery in humans. Infection with *S. flexneri* can result in more than a million deaths yearly and most of the victims are children in developing countries. Therefore, identifying novel and unique drug targets against this pathogen is instrumental to overcome the problem of drug resistance to the antibiotics given to patients as the current therapy. In this study, a comparative analysis of the metabolic pathways of the host and pathogen was performed to identify this pathogen’s essential enzymes for the survival and propose potential drug targets. First, we extracted the metabolic pathways of the host, *Homo sapiens*, and pathogen, *S.* flexneri, from the KEGG database. Next, we manually compared the pathways to categorize those that were exclusive to the pathogen. Further, all enzymes for the 26 unique pathways were extracted and submitted to the Geptop tool to identify essential enzymes for further screening in determining the feasibility of the therapeutic targets that were predicted and analyzed using PPI network analysis, subcellular localization, druggability testing, gene ontology and epitope mapping. Using these various criteria, we narrowed it down to prioritize 5 novel drug targets against *S. flexneri* and one vaccine drug targets against all strains of *Shigella*. Hence, we suggest the identified enzymes as the best putative drug targets for the effective treatment of *S. flexneri*.

## Introduction

*Shigella flexneri* is a gram-negative bacterium that belongs to the family *Enterobacteriaceae*. The genus *Shigella* can be classified into 4 species: *Shigella flexneri*, *Shigella boydii*, *Shigella sonnei* and *Shigella dysenteriae*. The species can be further diversified into different serotypes dependent on biochemical contrasts and variety of O-antigen. *S. flexneri* is separated into 13 serotypes. Shigellosis is an increasing cause of concern in developing nations as it causes more than 170 million estimated deaths each year, with the primary victims being children below 5 years of age [1]. *S. flexneri* is one of the most lethal organisms afflicting developing countries [2]. The mode of transmission is the fecal-oral route and a number as low as 10-100 can cause severe infections in human. *Shigella* species attack the epithelium of the colon and rectum in primates and people, causing the intense aggravation of the mucosa which is associated with shigellosis. Without compelling medications, shigellosis patients may develop auxiliary conditions, for example, septicaemia, pneumonia and haemolytic uremic disorder. The increased prevalence in developing countries is due to lack of food and water hygiene. The pathogenesis of *S. flexneri* is rapid in the system, as once it enters the colonial epithelium, it leads to very severe inflammation in the mucosal region. Traditional treatment of the disease condition begins with rehydration and antibiotics. However, there have been numerous reports in the last decade that the strain has become multi-drug resistant [3-6]. Over the last decade, *Shigella* has acquired plasmid-encoded resistance to the traditional drugs provided to patients as the first line of treatment. Hence, newer approaches and research must be undertaken to combat the increasing drug resistance.

With the advent of the post-genomic era, since the human genome project was completed successfully, there has been a revolution in the development of drug-designing approaches. The experimental approaches for drug designing are time consuming and costly. Host pathogen interactions can be studied by identifying the non-homologous proteins for host and pathogens. These proteins can be treated as targets in the drug discovery process. Pathway analysis using comparative methods has been the most sought-after approach in the last decade. A number of studies have been published in the past with reference to different pathogens on the basis of metabolic pathway analysis and protein-protein interaction studies [7-11]. However, an in-depth Gene Ontology analysis and epitope prediction to identify the putative targets have not been performed in previous work. These steps are essential because while Gene Ontology studies help us in identifying important features such as molecular function and cellular processes for a better understanding of the targets, epitope prediction helps us in identifying the main antigenic properties of the micro organisms. Our results indicate better reliability of the predicted targets for further validation by experimental studies. In the current study, we performed a metabolic pathway comparison between the pathogen and the host to identify essential enzymes for the survival of the bacteria and based on our prediction we have identified potential drug targets for the pathogen. The process began with the identification of metabolic pathways from the KEGG database for both the host and the pathogen. Next, we manually compared the pathways to identify those unique to the pathogen. Further, all enzymes for the unique pathways were extracted and submitted to an online tool for identification. The identified essential enzymes were further screened to determine the feasibility of therapeutic targets that were predicted and analyzed using novel drug target identification, cellular localization, gene ontology analysis and epitope prediction.

## Methodology

**Fig. 1:**
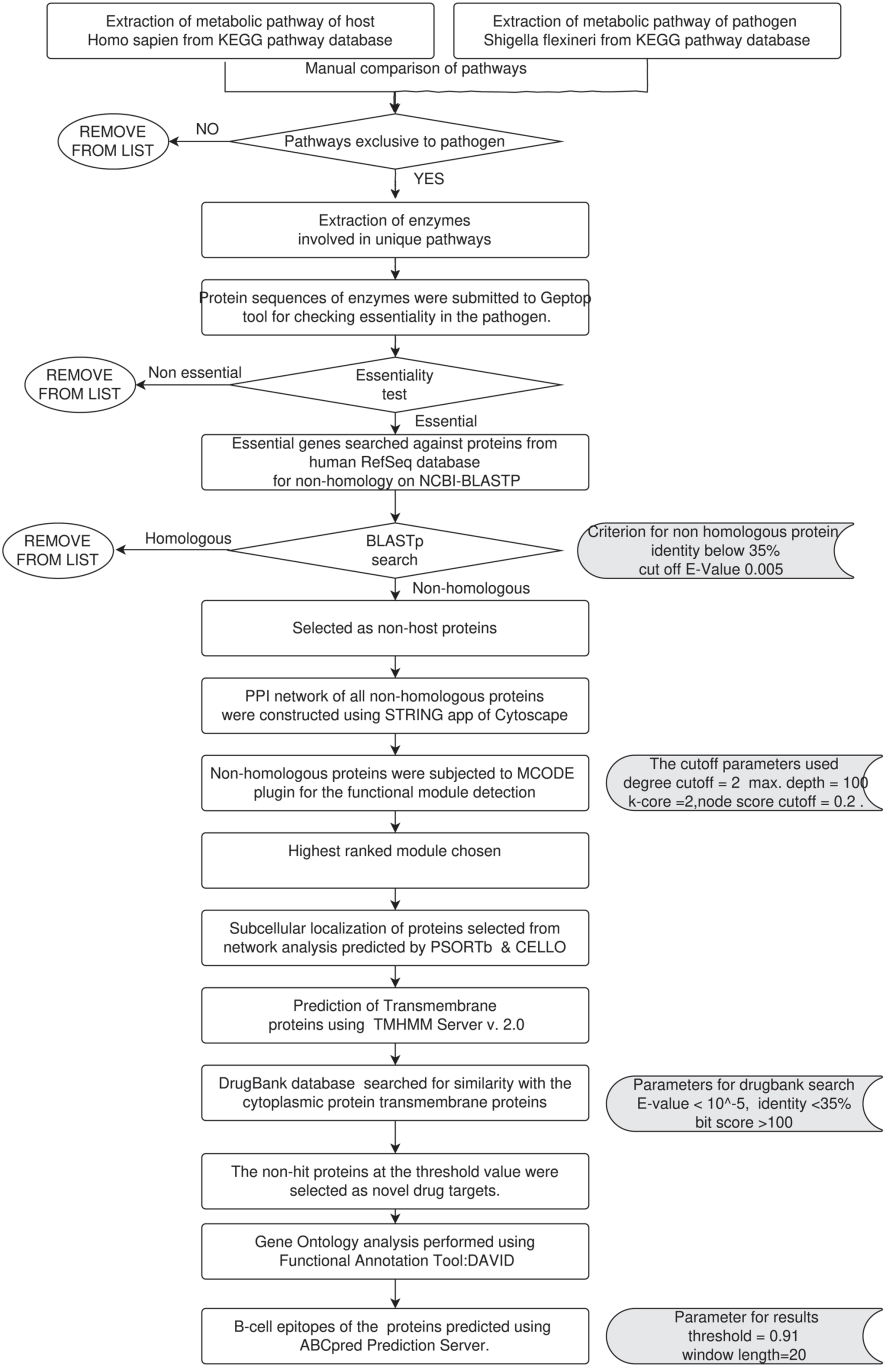
A schematic representation of the methodology.

A schematic representation of the methodology is given in Fig. 1.

### Comparative Analysis of Host and Pathogen Metabolic Pathways

The extraction of the metabolic pathways was done using the KEGG [12] pathway database for the host *Homo sapiens* and the pathogen *S. flexneri*. Pathways were then manually compared to identify those that were unique only to the pathogen. The criterion for selecting the pathways was based on whether the pathway was not present in the host. human but was present in the pathogen and unique to *S. flexneri*. The identification numbers of all metabolic pathways from both organisms were extracted from the database. A manual comparison was then conducted by placing the name of each individual pathway of the pathogen against the pathways of the host, *H. sapiens*. According to the KEGG database annotations, pathways that were absent in humans but did appear in the pathogen were considered unique to *S. flexneri*. Further, the enzymes that were involved in these unique pathways were extracted from the KEGG database and sequence retrieval was done from the NCBI database in the FASTA format.

### Identification of Non-Homologous Essential Genes

Protein sequences extracted in the FASTA format which were part of the unique pathways were submitted to the Geptop tool [13] to identify their essentiality in the pathogen. Geptop is a server used to identify essential genes of bacterial species by comparing their orthology and phylogeny with the essential gene database DEG. These essential genes were searched against proteins from the human RefSeq protein database for non-homology using NCBI-BLASTP [14]. Proteins having identity below 35% and an E-value cutoff of 0.005 were selected as non-host proteins.

### Protein Network Analysis

Functional interactions take place between genes/proteins and provide fundamental knowledge for cellular processing and systematic characterization, which play a vital role in molecular systems biology [15]. The PPI (Protein-Protein Interaction) network of all non-homologous proteins was built in Cytoscape v3.7.2 [16] using the STRING app. The interaction of network data was analyzed by network analyzer module [17]. The detection of the functional module of non-homologous proteins was done by MCODE plugin [18] under the degree cutoff = 2, maximum depth = 100, k-core = 2, and node score cutoff = 0.2.The uppermost hierarchical module was chosen as the utmost possible metabolic functional associations of the interacting proteins selected for further analysis.

### Subcellular Localization and Identification of Novel Drug Targets

Subcellular localization of the essential non-human proteins selected from network analysis was predicted by PSORTb v3.0.2 [19] and CELLO v2.518. Transmembrane proteins were identified by TMHMM Server v. 2.0. TMHMM server is based on the hidden Markov model. To find the most probable topology of a membrane protein, N-best algorithm was used. Proteins with a transmembrane helix predicted as having fewer than 50 amino acid residues from the N terminus were extracted as likely candidates for signal peptides. In addition, if a cleavage site was predicted with a probability > 0.5, the predicted signal peptide was cleaved off and the prediction was redone [20,21]. Only cytoplasmic and transmembrane proteins were selected as novel drug targets [23]. Further, the DrugBank databases [22] was used to identify novel targets amongst selected potential targets with an E-value of less than 10-5, sequence identity greater than 35%, and a bit score greater than 100.

### Gene Ontology and B-Cell Epitope Prediction

Novel drug targets of *Shigella flexneri* are ideal as drug candidates. Two-step methodology was performed. to analyze the drug targets identified through the work. As the first step, the DAVID Functional Annotation Tool was used for Gene Ontology analysis [24,25]. The complete list of 26 proteins identified was uploaded for Gene Ontology Analysis under the categories of Cellular Compartment (CC), Biological Process (BP), Molecular Function (MF), and InterPro Terms. Further, the B-cell epitopes of the proteins were predicted by ABCpred Prediction Server [26], threshold = 0.91, window length=20.

## Results

### Comparative Metabolic Pathway Analysis

Metabolic pathways of host and pathogen were examined using the KEGG database. Relative investigation was executed manually for the recognizable proof of pathways exclusive to *S. flexneri*. Metabolic pathways that are available in both the host and the pathogen are distinguished as normal pathways and those which are available as having pathogenicity and not in the host are deemed as novel pathways. A total of 85 metabolic pathways were extracted from the pathogen and among these 26 metabolic pathways (Table 1) were identified as unique to *S. flexneri*.

### Identification of Essential Genes

All the enzymes associated with these 26 unique pathways were identified and examined for their essentiality to the pathogen by using the tool Geptop 2.0. A total of 4179 genes are submitted and 395 of them are predicted as essential genes. Their accession no. and name were accessed from NCBI. BLASTP search was performed specifically against *H. sapiens* with an E-value threshold of 0.005 and an identity percentage of ≤ 35% were considered as non-host proteins which do not have human homologues. Out of 395 essential enzymes, 269 were found to be non-host as they did not show homology with human (Suppl. 1).

### Protein-Protein Interaction Network Analysis

**Fig. 2:**
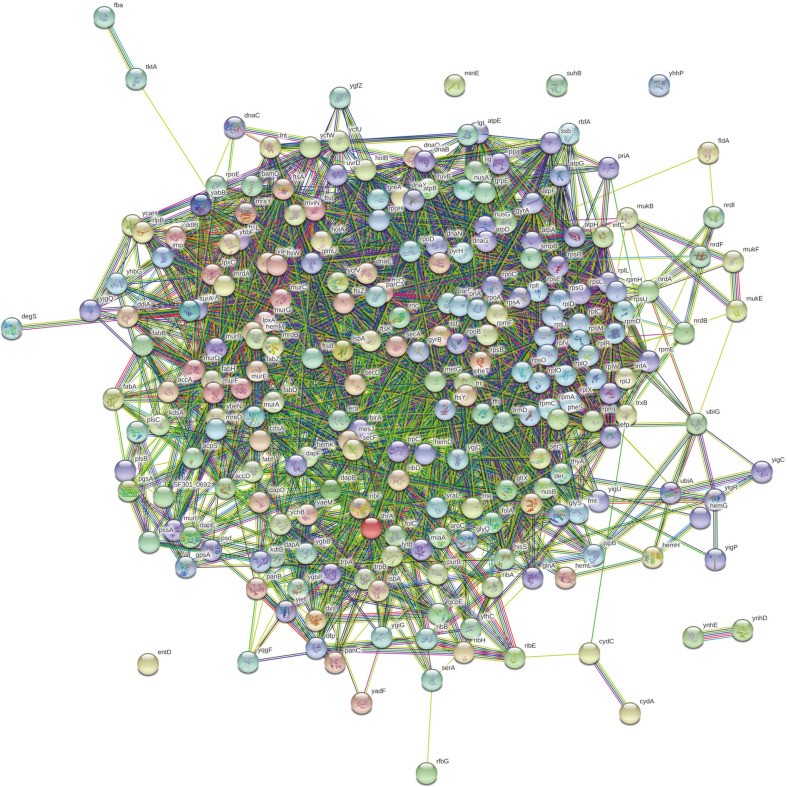
Protein-protein interaction network of non-host essential proteins from *Shigella flexneri*.

Functional associations between 269 non-host, essential genes of *S. flexneri* were studied using STRING app of Cytoscape v3.7.2 (Fig. 2). Interacting proteins with high confidence scores were visualized using MCODE plugin to predict a protein-protein complex data set. The molecular complex detection (MCODE) is a Cytoscape plugin that enabled detection of clusters in large protein interaction networks. Clusters obtained from the protein interaction network can be considered as protein complexes and functional modules, which can be identified as highly interconnected subgraphs [18]. Finding precisely the important interacting enzymes as network clusters provides insights into the exploration of potential drug targets. The highest ranked module having 57 nodes and 1444 edges was chosen as the utmost possible metabolic functional associations between identified proteins.

### Prediction of Subcellular Localization and Identification of Novel Drug Targets

Subcellular localization of 57 proteins revealed that 50 proteins were cytoplasmic, 4 were extracellular and 3 were transmembrane proteins (Fig. 2). Next, unveiling of novel targets was conducted using the DrugBank database. Proteins showing no matching hits against the DrugBank database at the threshold were nominated as novel drug targets. The results revealed 26 proteins that were uniquely involved in pathogen-specific unique pathways (Table 2).

### Gene Ontology and B-Cell Epitope Mapping

**Fig. 4:**
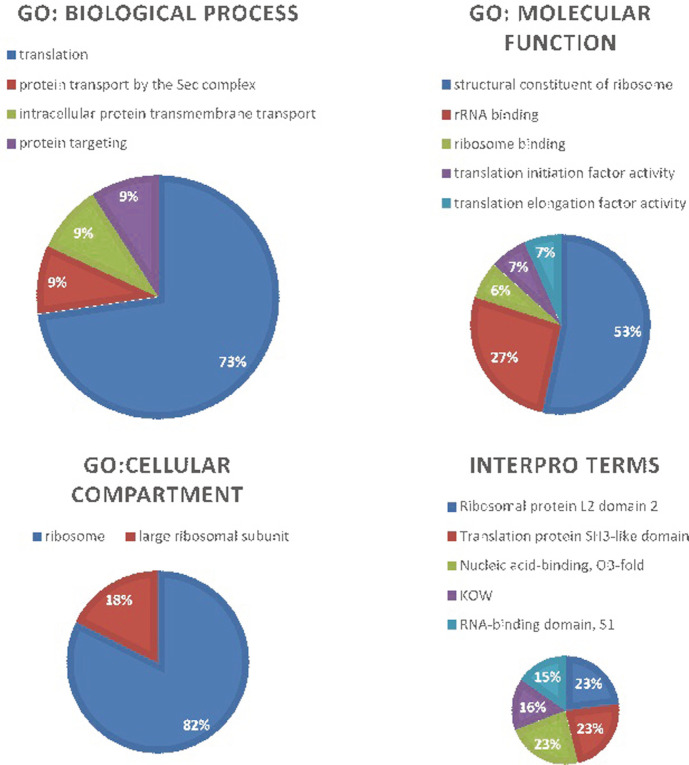
Gene Ontology analysis using Functional Annotation Tool: DAVID.

Gene Ontology analysis revealed interesting information on the drug proteins identified. Under the category of Biological Process, it was observed that 73% of the identified targets belonged to the “Translation” process. Gene Ontology for Molecular Function revealed that 53% of the proteins were associated with “structural constituent of ribosome”. Under the Cellular Component section, most of the drug targets belonged to the “ribosome” compartment. Further, performing an InterPro scan revealed an equal distribution amongst 4 major protein signatures: Ribosomal protein L2 domain 2, Translation protein SH3 like domain, Nucleic acid-binding domain, KOW and RNA-binding domain S1 (Fig. 4). In addition, B-cell epitope mapping was performed by ABCpred Prediction Server based on artificial neural network. The predicted B-cell epitopes were ordered based on their score attained. The top five highest ranked epitopes with a score of > 9.0 were selected as highest probable epitope (Table 3).

## Discussion

**Fig. 5:**
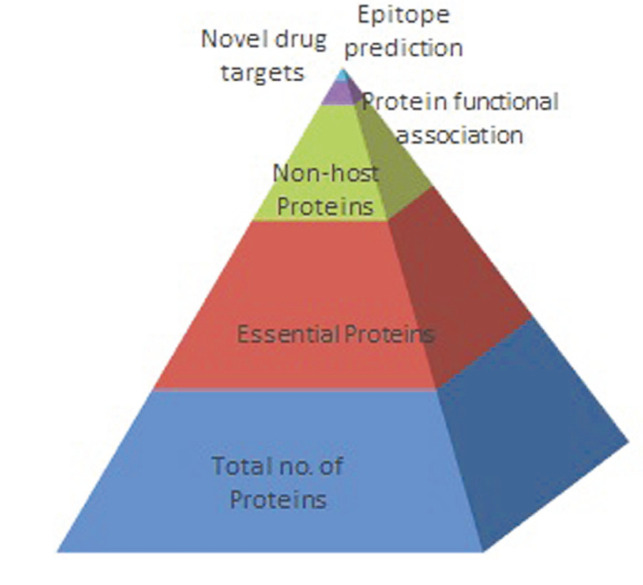
Subtractive dataset in *Shigella flexneri*.

The present study focused on subtractive genome analysis (Fig. 5) that led to identify the proteins which can be used as potential targets for drug development against the pathogenicity of *S. flexneri*. The rationale of picking targets using computational analysis depends on deciphering those proteins that are unique to the pathogen. Designing a drug exclusively for such a target will affect only the pathogen but won’t interfere with any aspects of the host biology. Therefore, using extensive in silico analysis, we identified broad-spectrum antibiotic targets and proposed elongation factor P, DNA-directed RNA polymerase subunit alpha, 30S ribosomal protein S1, 50S ribosomal protein L23, elongation factor Ts, cell division protein FtsQ as novel drug targets. The analysis revealed that protein elongation factor P, DNA-directed RNA polymerase subunit alpha, 30S ribosomal protein S1, cell division protein FtsQ, 50S ribosomal protein L23 and elongation factor Ts are cytoplasmic and can be used as potential drug targets against *S. flexneri*. However, protein cell division protein FtsQ is a membrane protein and can work as a suitable vaccine drug target against all strains of *Shigella*. Further, it has been observed that all these proteins have been involved significantly in the major metabolic pathways unique to the pathogen like lysine biosynthesis, lipopolysaccharide biosynthesis and peptidoglycan biosynthesis. Moreover, 30S ribosomal protein S1 is vital for binding with the mRNA in *Shigella*; thus, enabling recognition of the initiation point. The target protein is needed to translate mRNA with a short Shine-Dalgarno (SD) purine-rich sequence. Part of the 30S ribosomal subunit such as some nascent polypeptide chains can cross-link to this protein. Also, 50S ribosomal protein L23 is the underlying gathering protein and ties with the 23S rRNA. This protein target frames the center docking site as a requisite to bind with ribosome. One of the early assembly proteins, it binds 23S rRNA. It is an important target since it is one of the proteins that surrounds the polypeptide exit tunnel on the outside of the ribosome and forms the main docking site for trigger factor binding to the ribosome. It is an important contact for protein L29, and trigger factor when it is bound to the ribosome. The main function of DNA-directed RNA polymerase subunit alpha is to catalyze translation of DNA into RNA utilizing four ribonucleoside triphosphates as substrates. DNA-dependent RNA polymerase catalyzes the transcription of DNA into RNA using the four ribonucleoside triphosphates as substrates and is a homodimer. The RNAP catalytic core consists of 2 alpha, 1 beta, 1 beta' and 1 omega subunit. When a sigma factor is associated with the core the holoenzyme is formed, which can initiate transcription. The N-terminal domain is essential for RNAP assembly and basal transcription, whereas the C-terminal domain is involved in interaction with transcriptional regulators and with upstream promoter elements. Elongation factor P is mainly engaged with the synthesis of peptide bonds and reduces ribosome, slowing down protein which requires change of Lys-34. Elongation factor Ts is linked with the EF-Tu. GDP complex prompts the trading of GDP to GTP. It stays bound to the aminoacyl-tRNA.EF-Tu.GTP complex up to the GTP hydrolysis stage on the ribosome. Elongation factors are proteins that perform significant roles during the elongation cycle of protein biosynthesis on the ribosome. They alleviate ribosome pausing at polyproline (PPX) motifs by enabling peptide bond formation. In the absence of these proteins, PPX peptide bond formation can limit translation rate, leading to pleotropic phenotypes with slowed growth, increased antibiotic sensitivity, and loss of virulence. Hence, these proteins are the key targets of several antibiotics. Cell division protein FtsQ is a fundamental cell division protein. It performs a key role in the assembly of the divisome, a large protein complex that regulates bacterial cell division. It formulates a stable trimeric complex with FtsB and FtsL, which is essential for successful cell division [27, 28]. The interactions with FtsB and FtsL mostly take place in the periplasmic domains of these proteins, indicating that drugs targeting these interactions would only have to cross the outer membrane of the bacterium. Hence it can be suggested as a potential vaccine candidate for antibacterial treatment [29, 30].

## Conclusions

*Shigella flexneri* is a gram-negative bacterium that is a member of the family *Enterobacteriaceae*. It is the most endemic species and the most lethal for developing countries. Traditional treatment of the disease condition begins with rehydration and antibiotics. However, there have been numerous reports in the last decade suggesting that the strain has become multi-drug resistant. Over the last decade, *Shigella* has acquired plasmid-encoded resistance to the traditional drugs that were provided to patients as the first line of treatment. Here, we employed comparative metabolic pathway analysis for the identification of essential enzymes for the survival of the bacteria and based on our prediction we are able to propose potential drug targets for the pathogen. In conclusion, we can suggest five essential enzymes, elongation factor P, DNA-directed RNA polymerase subunit alpha, 30S ribosomal protein S1, 50S ribosomal protein L23 and elongation factor Ts as the best putative drug targets against the pathogenesis of *Shigella flexneri* and cell division protein FtsQ as the vaccine drug targets against all strains of *Shigella* . To the best of our understanding, the research outcome from our work could highlight a novel approach and identify drug targets to overcome the infection caused by the pathogen. In the future, whole genome proteome sequence data can be retrieved for all the strains of *Shigella* and further core proteomic analysis can be performed for the dataset, leading to improved and more enhanced applications of this work.

## Supplemental Materials



Supplementary data for this paper are available on-line only at http://jmb.or.kr.

## Figures and Tables

**Fig. 3:**
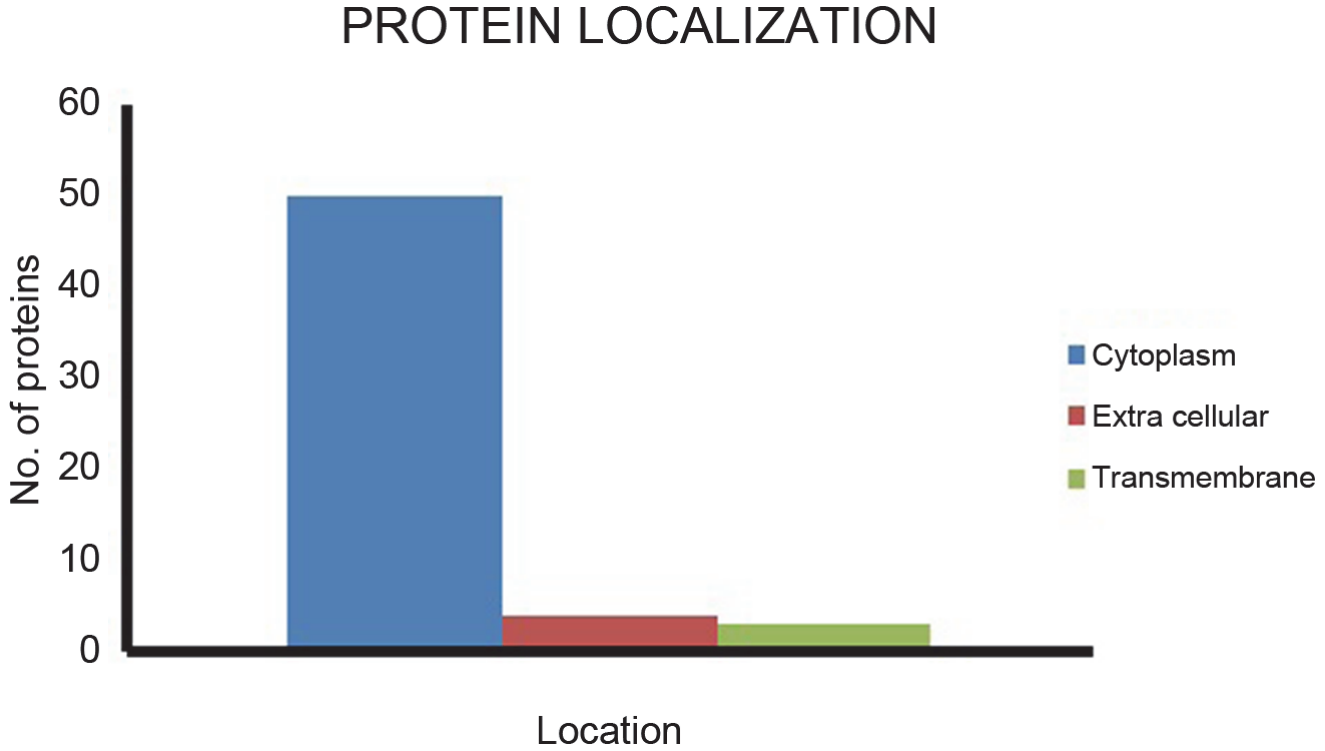
Prediction of subcellular localization.

**Table 1:** List of metabolic pathways unique to *Shigella flexneri*.

S. No.	Pathway ID	Pathway name
1	00660	C5-Branched dibasic acid metabolism
2	00680	Methane metabolism
3	00121	Secondary bile acid biosynthesis
4	00300	Lysine biosynthesis
5	00460	Cyanoamino acid metabolism
6	00473	D-Alanine metabolism
7	00540	Lipopolysaccharide biosynthesis
8	00550	Peptidoglycan biosynthesis
9	00903	Limonene and pinene degradation
10	00281	Geraniol degradation
11	00523	Polyketide sugar unit biosynthesis
12	01053	Biosynthesis of siderophore group nonribosomal peptides
13	00332	Carbapenem biosynthesis
14	00261	Monobactam biosynthesis
15	00521	Streptomycin biosynthesis
16	00525	Acarbose and validamycin biosynthesis
17	00401	Novobiocin biosynthesis
18	00362	Benzoate degradation
19	00627	Aminobenzoate degradation
20	00364	Fluorobenzoate degradation
21	00625	Chloroalkane and chloroalkene degradation
22	00361	Chlorocyclohexane and chlorobenzene degradation
23	00623	Toluene degradation
24	00633	Nitrotoluene degradation
25	00930	Caprolactam degradation
26	00626	Naphthalene degradation

**Table 2:** List of proteins selected as novel drug targets.

S No.	Accession no.	Protein names	Subcellular localization	Novel drug targets
1	NP_710014	Elongation factor P (EF-P)	Cytoplasm	No Hits
2	NP_706048	Cell division protein FtsQ	Transmembrane	No Hits
3	NP_706769	Translation initiation factor IF-1	Cytoplasm	No Hits
4	NP_707396	Translation initiation factor IF-3	Cytoplasm	No Hits
5	NP_709777	Transcription termination	Cytoplasm	No Hits
6	NP_709088	Protein translocase subunit SecY	Transmembrane	No Hits
7	NP_710066	50S ribosomal protein L9	Cytoplasm	No Hits
8	NP_709089	50S ribosomal protein L15	Cytoplasm	No Hits
9	NP_709082	50S ribosomal protein L17	Cytoplasm	No Hits
10	NP_709092	50S ribosomal protein L18	Cytoplasm	No Hits
11	NP_708985	50S ribosomal protein L21	Cytoplasm	No Hits
12	NP_709106	50S ribosomal protein L23	Cytoplasm	No Hits
13	NP_709097	50S ribosomal protein L24	Cytoplasm	No Hits
14	NP_709416	50S ribosomal protein L28	Cytoplasm	No Hits
15	NP_709100	50S ribosomal protein L29	Cytoplasm	No Hits
16	NP_709090	50S ribosomal protein L30	Cytoplasm	No Hits
17	NP_709740	50S ribosomal protein L31	Cytoplasm	No Hits
18	NP_707005	50S ribosomal protein L32	Cytoplasm	No Hits
19	NP_709497	50S ribosomal protein L34	Cytoplasm	No Hits
20	NP_707397	50S ribosomal protein L35	Cytoplasm	No Hits
21	NP_709083	DNA-directed RNA polymerase subunit alpha	Cytoplasm	No Hits
22	NP_706830	30S ribosomal protein S1	Cytoplasm	No Hits
23	NP_708875	30S ribosomal protein S21	Cytoplasm	No Hits
24	NP_709776	Protein translocase subunit SecE	Transmembrane	No Hits
25	NP_706115	Elongation factor Ts (EF-Ts)	Cytoplasm	No Hits
26	NP_708456	Ribosome maturation factor RimM	Cytoplasm	No Hits

**Table 3:** List of predicted epitopes having score value greater than threshold.

Rank	Accession no.	Protein Name	Sequence	Start position	Score
1	NP_710014	Elongation factor P (EF-P)	KVPLFVQIGEVIKVDTRSGE	199	0.96
2	NP_709083	DNA-directed RNA polymerase subunit alpha	VILTLNKSGIGPVTAADITH	3643	0.94
3	NP_706830	30S ribosomal protein S1	VTGVINGKVKGGFTVELNGI	4018	0.93
4	NP_706048	Cell division protein FtsQ	AAMTARRSWQLTLNNDIKLN	458	0.92
4	NP_709106	50S ribosomal protein L23	STAMEKSNTIVLKVAKDATK	2582	0.92
5	NP_706115	Elongation factor Ts (EF-Ts)	NMRKSGAIKAAKKAGNVAAD	4827	0.91
