# Supplementary material for: Computational Identification of Essential Enzymes as Potential Drug Targets in Shigella flexneri Pathogenesis Using Metabolic Pathway Analysis and Epitope Mapping
Source: J Microbiol Biotechnol. 2020 Dec 14;31(4):621–9. doi: 10.4014/jmb.2007.07006 (PMC9723279; doi:10.4014/jmb.2007.07006)
Supplement: Supplementary file 1 [file jmb-31-4-621-supple.pdf]

S.No.	Essential genes from Geptop result			Accession no	Name	Non homologous
1	1	0.202	gi|56479580|ref|NP_7	NP_705962	bifunctional aspartokinase I/homoserine dehydrogenase I	yes
2	1	0.3335	gi|24111463|ref|NP_7	NP_705973	molecular chaperone DnaK	no
3	1	0.1976	gi|56479584|ref|NP_7	NP_705974	molecular chaperone DnaJ	no
4	1	0.3103	gi|344915203|ref|NP_	WP_011069241	30S ribosomal protein S20	Yes
5	1	0.3841	gi|24111470|ref|NP_7	NP_705980	bifunctional riboflavin kinase/FMN adenylyltransferase	yes
6	1	0.4271	gi|24111471|ref|NP_7	NP_705981	isoleucyl-tRNA synthetase	no
7	1	0.3378	gi|24111472|ref|NP_7	NP_705982	lipoprotein signal peptidase	yes
8	1	0.3394	gi|56479587|ref|NP_7	NP_705985	4-hydroxy-3-methylbut-2-enyl diphosphate reductase	yes
9	1	0.363	gi|24111477|ref|NP_7	NP_705987	4-hydroxy-tetrahydrodipicolinate reductase	yes
10	1	0.4395	gi|56479590|ref|NP_7	NP_706004	dihydrofolate reductase	yes
11	1	0.1877	gi|24111499|ref|NP_7	NP_706009	SurA [	yes
12	1	0.3012	gi|24111500|ref|NP_7	NP_706010	LPS assembly outer membrane complex protein LptD	yes
13	1	0.1833	gi|56479598|ref|NP_7	NP_706036	cell division protein MraZ	yes
14	1	0.2145	gi|24111527|ref|NP_7	NP_706037	16S rRNA m(4)C1402 methyltransferase	no
15	1	0.262	gi|24111528|ref|NP_7	NP_706038	cell division protein FtsL	yes
16	1	0.3721	gi|24111529|ref|NP_7	NP_706039	peptidoglycan synthase FtsI	yes
17	1	0.3953	gi|24111530|ref|NP_7	NP_706040	UDP-N-acetylmuramoylalanyl-D-glutamate--2,6-diaminopimelate ligase	yes
18	1	0.449	gi|24111531|ref|NP_7	NP_706041	UDP-N-acetylmuramoyl-tripeptide--D-alanyl-D-alanine ligase	yes
19	1	0.4284	gi|24111532|ref|NP_7	NP_706042	phospho-N-acetylmuramoyl-pentapeptide-transferase	yes
20	1	0.439	gi|24111533|ref|NP_7	NP_706043	UDP-N-acetylmuramoyl-L-alanyl-D-glutamate synthetase	yes
21	1	0.3737	gi|56479599|ref|NP_7	NP_706044	cell division protein FtsW	yes
22	1	0.4803	gi|24111535|ref|NP_7	NP_706045	undecaprenyldiphospho-muramoylpentapeptide beta-N-acetylglucosaminyltransferase	yes
23	1	0.3752	gi|24111536|ref|NP_7	NP_706046	ligase	yes
24	1	0.1865	gi|24111537|ref|NP_7	NP_706047	D-alanine--D-alanine ligase	yes
25	1	0.314	gi|24111538|ref|NP_7	NP_706048	cell division protein FtsQ	yes
26	1	0.4407	gi|24111539|ref|NP_7	NP_706049	cell division protein FtsA	yes
27	1	0.5165	gi|56479600|ref|NP_7	NP_706050	cell division protein FtsZ	yes
28	1	0.2595	gi|24111541|ref|NP_7	NP_706051	UDP-3-O-[3-hydroxymyristoyl] N-acetylglucosamine deacetylase	yes
29	1	0.4489	gi|24111543|ref|NP_7	NP_706053	preprotein translocase subunit SecA	yes
30	1	0.3478	gi|24111548|ref|NP_7	NP_706058	dephospho-CoA kinase	no
31	1	0.2847	gi|56479605|ref|NP_7	NP_706070	dihydrolipoamide dehydrogenase	no
32	1	0.1716	gi|24111569|ref|NP_7	NP_706079	carbonic anhydrase	yes
33	1	0.1703	gi|24111576|ref|NP_7	NP_706086	pantoate--beta-alanine ligase	yes
34	1	0.1703	gi|24111577|ref|NP_7	NP_706087	3-methyl-2-oxobutanoate hydroxymethyltransferase	yes
35	1	0.2714	gi|24111580|ref|NP_7	NP_706090	2-amino-4-hydroxy-6-hydroxymethyldihydropteridine pyrophosphokinase	no
36	1	0.2305	gi|24111592|ref|NP_7	NP_706102	glutamate-1-semialdehyde aminotransferase	yes
37	1	0.2606	gi|56479616|ref|NP_7	NP_706104	iron-sulfur cluster insertion protein ErpA	no
38	1	0.26	gi|24111601|ref|NP_7	NP_706111	2,3,4,5-tetrahydropyridine-2,6-dicarboxylate N-succinyltransferase	yes
39	1	0.4493	gi|24111603|ref|NP_7	NP_706113	methionine aminopeptidase	no
40	1	0.4891	gi|56479617|ref|NP_7	NP_706114	30S ribosomal protein S2	yes
41	1	0.4589	gi|24111605|ref|NP_7	NP_706115	elongation factor Ts	yes
42	1	0.48	gi|24111606|ref|NP_7	NP_706116	uridylate kinase	yes
43	1	0.4699	gi|24111607|ref|NP_7	NP_706117	ribosome recycling factor	yes
44	1	0.3394	gi|24111608|ref|NP_7	NP_706118	1-deoxy-D-xylulose 5-phosphate reductoisomerase	yes
45	1	0.3965	gi|24111609|ref|NP_7	NP_706119	undecaprenyl pyrophosphate synthase	no

46	1	0.4595	gi|344915214|ref|NP_	NP_706120	CDP-diglyceride synthetase	yes
47	1	0.2314	gi|56479619|ref|NP_7	NP_706121	zinc metallopeptidase RseP	no
48	1	0.3262	gi|24111612|ref|NP_7	NP_706122	outer membrane protein assembly factor YaeT	yes
49	1	0.314	gi|24111614|ref|NP_7	NP_706124	UDP-3-O-[3-hydroxymyristoyl] glucosamine N-acyltransferase	no
50	1	0.3862	gi|56479620|ref|NP_7	NP_706125	(3R)-hydroxymyristoyl-ACP dehydratase	yes
51	1	0.3257	gi|24111616|ref|NP_7	NP_706126	acyl-[acyl-carrier-protein]--UDP-N-acetylglucosamine O-acyltransferase	yes
52	1	0.2873	gi|56479621|ref|NP_7	NP_706127	lipid-A-disaccharide synthase	no
53	1	0.4891	gi|24111619|ref|NP_7	NP_706129	DNA polymerase III subunit alpha	yes
54	1	0.4608	gi|24111620|ref|NP_7	NP_706130	acetyl-CoA carboxylase carboxyltransferase subunit alpha	yes
55	1	0.4281	gi|24111623|ref|NP_7	NP_706133	tRNA(Ile)-lysidine synthetase	yes
56	1	0.4701	gi|56479623|ref|NP_7	NP_706140	prolyl-tRNA synthetase	no
57	1	0.2165	gi|56479626|ref|NP_7	NP_706156	DNA polymerase III subunit epsilon	yes
58	1	0.2564	gi|24111677|ref|NP_7	NP_706187	D-alanyl-alanine synthetase A	yes
59	1	0.2584	gi|24111693|ref|NP_7	NP_706203	delta-aminolevulinic acid dehydratase	no
60	1	0.1534	gi|24111785|ref|NP_7	NP_706295	preprotein translocase subunit YajC	no
61	1	0.2306	gi|24111786|ref|NP_7	NP_706296	preprotein translocase subunit SecD	yes
62	1	0.3739	gi|56479648|ref|NP_7	NP_706297	preprotein translocase subunit SecF	yes
63	1	0.2587	gi|24111792|ref|NP_7	NP_706302	bifunctional diaminohydroxyphosphoribosylaminopyrimidine deaminase/5-amino-6-(5-phosphoribosylamino)uracil reductase	yes
64	1	0.2015	gi|56479650|ref|NP_7	NP_706303	6,7-dimethyl-8-ribityllumazine synthase	yes
65	1	0.1845	gi|24111794|ref|NP_7	NP_706304	transcription antitermination protein NusB	yes
66	1	0.2871	gi|24111795|ref|NP_7	NP_706305	thiamine monophosphate kinase	no
67	1	0.4197	gi|24111798|ref|NP_7	NP_706308	1-deoxy-D-xylulose-5-phosphate synthase	yes
68	1	0.2877	gi|24111799|ref|NP_7	NP_706309	geranyltranstransferase	yes
69	1	0.1712	gi|56479669|ref|NP_7	NP_706362	adenine phosphoribosyltransferase	no
70	1	0.4484	gi|24111853|ref|NP_7	NP_706363	DNA polymerase III subunits gamma and tau	yes
71	1	0.459	gi|56479671|ref|NP_7	NP_706367	adenylate kinase	no
72	1	0.3753	gi|24111858|ref|NP_7	NP_706368	ferrochelatase	yes
73	1	0.3009	gi|24111892|ref|NP_7	NP_706402	UDP-2,3-diacylglucosamine hydrolase	yes
74	1	0.4489	gi|24111894|ref|NP_7	NP_706404	cysteinyl-tRNA synthetase	no
75	1	0.3719	gi|24111897|ref|NP_7	NP_706407	bifunctional 5,10-methylene-tetrahydrofolate dehydrogenase/ 5,10-methylene-tetrahydrofolate cyclohydrolase	no
76	1	0.1866	gi|56479681|ref|NP_7	NP_706438	[illegible] complex phosphopantetheinyltransferase	yes
77	1	0.1575	gi|24111991|ref|NP_7	NP_706501	cytochrome d terminal oxidase subunit 1	yes
78	1	0.296	gi|24111997|ref|NP_7	NP_706507	dihydrolipoamide succinyltransferase	no
79	1	0.1847	gi|24111998|ref|NP_7	NP_706508	E1	no
80	1	0.2332	gi|56479700|ref|NP_7	NP_706540	flavodoxin FldA	yes
81	1	0.3276	gi|24112034|ref|NP_7	NP_706544	glutaminyl-tRNA synthetase	no
82	1	0.325	gi|24112043|ref|NP_7	NP_706553	metal-binding heat shock protein	no
83	1	0.2311	gi|24112045|ref|NP_7	NP_706555	apolipoprotein N-acyltransferase	yes
84	1	0.4278	gi|56479706|ref|NP_7	NP_706568	leucyl-tRNA synthetase	no
85	1	0.2339	gi|24112059|ref|NP_7	NP_706569	LPS-assembly lipoprotein RlpB	yes
86	1	0.3276	gi|24112060|ref|NP_7	NP_706570	DNA polymerase III subunit delta	yes
87	1	0.335	gi|24112061|ref|NP_7	NP_706571	nicotinic acid mononucleotide adenylyltransferase	yes
88	1	0.2333	gi|24112065|ref|NP_7	NP_706575	penicillin-binding protein 2	yes
89	1	0.2316	gi|24112066|ref|NP_7	NP_706576	cell wall shape-determining protein	yes
90	1	0.2013	gi|344915230|ref|NP_	NP_706580	lipoate biosynthesis protein LipB	no
91	1	0.1689	gi|24112072|ref|NP_7	NP_706582	lipoyl synthase	no
92	1	0.4891	gi|24112259|ref|NP_7	NP_706769	translation initiation factor IF-1	yes
93	1	0.1728	gi|24112261|ref|NP_7	NP_706771	cysteine/glutathione ABC transporter membrane/ATP-binding protein	yes
94	1	0.282	gi|56479756|ref|NP_7	NP_706773	thioredoxin reductase	yes
95	1	0.2172	gi|24112265|ref|NP_7	NP_706775	DNA translocase FtsK	yes
96	1	0.3145	gi|24112266|ref|NP_7	NP_706776	lipoprotein chaperone	no
97	1	0.4879	gi|24112268|ref|NP_7	NP_706778	seryl-tRNA synthetase	yes

98	1	0.3954	gi|24112319|ref|NP_7	NP_706829	cytidylate kinase	no
99	1	0.3138	gi|24112320|ref|NP_7	NP_706830	30S ribosomal protein S1	yes
100	1	0.3504	gi|24112322|ref|NP_7	NP_706832	lipid transporter ATP-binding/permease	no
101	1	0.3146	gi|24112323|ref|NP_7	NP_706833	tetraacyldisaccharide 4'-kinase	yes
102	1	0.1865	gi|24112325|ref|NP_7	NP_706835	hypothetical protein SF0913	no
103	1	0.3275	gi|24112326|ref|NP_7	NP_706836	3-deoxy-manno-octulosonate cytidylyltransferase	no
104	1	0.1869	gi|24112330|ref|NP_7	NP_706840	condesin subunit F	yes
105	1	0.1729	gi|56479772|ref|NP_7	NP_706841	condesin subunit E	yes
106	1	0.1729	gi|24112332|ref|NP_7	NP_706842	**cell division protein MukB**	yes
107	1	0.349	gi|56479774|ref|NP_7	NP_706849	asparaginyl-tRNA synthetase	no
108	1	0.2616	gi|24112366|ref|NP_7	NP_706876	**3-hydroxydecanoyl-ACP dehydratase**	yes
109	1	0.1886	gi|344915242|ref|NP_	NP_706892	sulfite reductase	no
110	1	0.3502	gi|56479815|ref|NP_7	NP_706990	virulence factor	yes
111	1	0.2316	gi|344915246|ref|NP_	NP_707000	RNase E	yes
112	1	0.2004	gi|24112495|ref|NP_7	NP_707005	50S ribosomal protein L32	yes
113	1	0.2833	gi|344915248|ref|NP_	NP_707006	PlsX protein	no
114	1	0.2453	gi|24112497|ref|NP_7	NP_707007	3-oxoacyl-ACP synthase III	yes
115	1	0.4188	gi|24112498|ref|NP_7	NP_707008	malonyl CoA-ACP transacylase	yes
116	1	0.4608	gi|24112499|ref|NP_7	NP_707009	3-ketoacyl-ACP reductase	no
117	1	0.4282	gi|24112500|ref|NP_7	NP_707010	acyl carrier protein	no
118	1	0.2272	gi|24112501|ref|NP_7	NP_707011	3-oxoacyl-ACP synthase	no
119	1	0.4603	gi|24112504|ref|NP_7	NP_707014	thymidylate kinase	no
120	1	0.2334	gi|24112505|ref|NP_7	NP_707015	DNA polymerase III subunit delta'	yes
121	1	0.2045	gi|56479828|ref|NP_7	NP_707031	outer membrane-specific lipoprotein transporter subunit LolC	yes
122	1	0.2889	gi|24112522|ref|NP_7	NP_707032	lipoprotein transporter ATP-binding subunit	yes
123	1	0.3146	gi|24112523|ref|NP_7	NP_707033	outer membrane-specific lipoprotein transporter subunit LolE	yes
124	1	0.2873	gi|24112550|ref|NP_7	NP_707060	adenylosuccinate lyase	yes
125	1	0.3726	gi|344915250|ref|NP_	NP_707062	hypothetical protein SF1152	no
126	1	0.1881	gi|24112560|ref|NP_7	NP_707070	cell division topological specificity factor MinE	yes
127	1	0.4585	gi|24112603|ref|NP_7	NP_707113	peptidyl-tRNA hydrolase	no
128	1	0.4484	gi|56479854|ref|NP_7	NP_707116	ribose-phosphate pyrophosphokinase	no
129	1	0.3389	gi|24112607|ref|NP_7	NP_707117	4-diphosphocytidyl-2C-methyl-D-erythritol kinase	yes
130	1	0.2889	gi|24112608|ref|NP_7	NP_707118	molecular chaperone LolB	yes
131	1	0.2589	gi|56479855|ref|NP_7	NP_707119	glutamyl-tRNA reductase	no
132	1	0.4891	gi|24112610|ref|NP_7	NP_707120	peptide chain release factor 1	no
133	1	0.2315	gi|24112611|ref|NP_7	NP_707121	methionine-dependent methyltransferase	yes
134	1	0.2321	gi|24112614|ref|NP_7	NP_707124	2-dehydro-3-deoxyphosphooctonate aldolase	yes
135	1	0.1678	gi|24112632|ref|NP_7	NP_707142	UTP-glucose-1-phosphate uridylyltransferase	no
136	1	0.1704	gi|24112660|ref|NP_7	NP_707170	tryptophan synthase subunit beta	yes
137	1	0.3947	gi|56479869|ref|NP_7	NP_707181	DNA topoisomerase I subunit omega	no
138	1	0.2886	gi|56479870|ref|NP_7	NP_707186	GTP cyclohydrolase II	yes
139	1	0.35	gi|24112687|ref|NP_7	NP_707197	enoyl-ACP reductase	yes
140	1	0.4183	gi|24112805|ref|NP_7	NP_707315	hypothetical protein SF1421	no
141	1	0.3117	gi|56479896|ref|NP_7	NP_707335	glyceraldehyde-3-phosphate dehydrogenase	no
142	1	0.3835	gi|24112861|ref|NP_7	NP_707371	NAD synthetase	no
143	1	0.4984	gi|24112885|ref|NP_7	NP_707395	threonyl-tRNA synthetase	no
144	1	0.4886	gi|24112886|ref|NP_7	NP_707396	translation initiation factor IF-3	yes
145	1	0.3954	gi|56479920|ref|NP_7	NP_707397	50S ribosomal protein L35	yes
146	1	0.4699	gi|24112888|ref|NP_7	NP_707398	50S ribosomal protein L20	no
147	1	0.4891	gi|24112890|ref|NP_7	NP_707400	phenylalanyl-tRNA synthetase subunit alpha	yes
148	1	0.4984	gi|24112891|ref|NP_7	NP_707401	phenylalanyl-tRNA synthetase subunit beta	yes
149	1	0.4485	gi|56479945|ref|NP_7	NP_707537	tyrosyl-tRNA synthetase	no
150	1	0.1868	gi|24113028|ref|NP_7	NP_707538	pyridoxamine 5'-phosphate oxidase	no
151	1	0.2328	gi|24113052|ref|NP_7	NP_707562	riboflavin synthase subunit alpha	yes
152	1	0.2266	gi|56479951|ref|NP_7	NP_707575	pyruvate kinase	no

153	1	0.1983	gi|24113071|ref|NP_7	NP_707581	ABC transporter ATP-binding protein	yes
154	1	0.1683	gi|24113072|ref|NP_7	NP_707582	cysteine desulfurase	yes
155	1	0.2282	gi|24113210|ref|NP_7	NP_707720	Holliday junction DNA helicase RuvB	yes
156	1	0.4177	gi|24113215|ref|NP_7	NP_707725	aspartyl-tRNA synthetase	no
157	1	0.405	gi|24113254|ref|NP_7	NP_707764	arginyl-tRNA synthetase	no
158	1	0.4892	gi|24113289|ref|NP_7	NP_707799	synthetase	yes
159	1	0.1556	gi|24113420|ref|NP_7	NP_707930	dTDP-rhamnosyl transferase	yes
160	1	0.2826	gi|56480034|ref|NP_7	NP_707984	tagatose-bisphosphate aldolase	no
161	1	0.1535	gi|56480037|ref|NP_7	NP_708000	antiporter inner membrane protein	no
162	1	0.459	gi|56480038|ref|NP_7	NP_708001	methionyl-tRNA synthetase	yes
163	1	0.3121	gi|24113542|ref|NP_7	NP_708052	GTP cyclohydrolase I	no
164	1	0.1864	gi|56480061|ref|NP_7	NP_708084	50S ribosomal protein L25	no
165	1	0.438	gi|24113610|ref|NP_7	NP_708120	DNA gyrase subunit A	yes
166	1	0.2164	gi|24113611|ref|NP_7	NP_708121	bifunctional 3-demethylubiquinone-9 3-methyltransferase/ 2-octaprenyl-6-hydroxy phenol methylase	yes
167	1	0.262	gi|24113614|ref|NP_7	NP_708124	ribonucleotide-diphosphate reductase subunit alpha	yes
168	1	0.3146	gi|344915291|ref|NP_	NP_708125	ribonucleoside-diphosphate reductase 1 subunit beta	yes
169	1	0.1698	gi|344915295|ref|NP_	NP_708154	hypothetical protein SF2347	no
170	1	0.2865	gi|24113687|ref|NP_7	NP_708197	bifunctional folylpolyglutamate synthase/ dihydrofolate synthase	yes
171	1	0.4297	gi|56480089|ref|NP_7	NP_708198	acetyl-CoA carboxylase carboxyltransferase subunit beta	yes
172	1	0.2451	gi|24113695|ref|NP_7	NP_708205	3-oxoacyl-ACP synthase	yes
173	1	0.1704	gi|56480091|ref|NP_7	NP_708211	chorismate synthase	yes
174	1	0.4891	gi|24113752|ref|NP_7	NP_708262	glutamyl-tRNA synthetase	yes
175	1	0.5169	gi|24113756|ref|NP_7	NP_708266	NAD-dependent DNA ligase LigA	yes
176	1	0.2045	gi|56480101|ref|NP_7	NP_708267	cell division protein ZipA	no
177	1	0.1983	gi|24113787|ref|NP_7	NP_708297	glutamate racemase	yes
178	1	0.3001	gi|24113800|ref|NP_7	NP_708310	desuccinylase	yes
179	1	0.3377	gi|56480116|ref|NP_7	NP_708317	synthase	yes
180	1	0.2293	gi|24113836|ref|NP_7	NP_708346	GMP synthase	no
181	1	0.1992	gi|56480121|ref|NP_7	NP_708347	dehydrogenase	no
182	1	0.4778	gi|56480123|ref|NP_7	NP_708350	GTP-binding protein Der	yes
183	1	0.4577	gi|24113843|ref|NP_7	NP_708353	histidyl-tRNA synthetase	yes
184	1	0.3131	gi|24113844|ref|NP_7	NP_708354	4-hydroxy-3-methylbut-2-en-1-yl diphosphate synthase (flavodoxin)	yes
185	1	0.2574	gi|24113858|ref|NP_7	NP_708368	scaffold protein	no
186	1	0.2975	gi|56480127|ref|NP_7	NP_708369	cysteine desulfurase	no
187	1	0.1712	gi|24113862|ref|NP_7	NP_708372	inositol monophosphatase	yes
188	1	0.2701	gi|56480132|ref|NP_7	NP_708388	serine hydroxymethyltransferase	no
189	1	0.3141	gi|24113886|ref|NP_7	NP_708396	tRNA-specific adenosine deaminase	yes
190	1	0.3604	gi|24113905|ref|NP_7	NP_708415	4'-phosphopantetheinyl transferase	yes
191	1	0.4083	gi|24113908|ref|NP_7	NP_708418	GTPase Era	yes
192	1	0.2449	gi|24113909|ref|NP_7	NP_708419	ribonuclease III	yes
193	1	0.3387	gi|24113910|ref|NP_7	NP_708420	signal peptidase I	no
194	1	0.2025	gi|56480143|ref|NP_7	NP_708425	RNA polymerase sigma factor RpoE	yes
195	1	0.2179	gi|56480147|ref|NP_7	NP_708436	phosphatidylserine synthase	yes
196	1	0.2321	gi|24113931|ref|NP_7	NP_708441	BamD	yes
197	1	0.1551	gi|56480150|ref|NP_7	NP_708444.2	protein disaggregation chaperone	no
198	1	0.4289	gi|24113944|ref|NP_7	NP_708454.1	50S ribosomal protein L19	no
199	1	0.4588	gi|24113945|ref|NP_7	NP_708455.1	tRNA (guanine-N(1)-)-methyltransferase	yes
200	1	0.3603	gi|56480152|ref|NP_7	NP_708456.2	16S rRNA-processing protein RimM	yes
201	1	0.4295	gi|24113947|ref|NP_7	NP_057149.1	28S ribosomal protein S16, mitochondrial [Homo sapiens]	No
202	1	0.4273	gi|24113948|ref|NP_7	NP_708458.1	signal recognition particle protein	Yes
203	1	0.4179	gi|24113951|ref|NP_7	NP_708461.1	heat shock protein GrpE	YEs
204	1	0.3841	gi|24113952|ref|NP_7	NP_708462.1	inorganic polyphosphate/ATP-NAD kinase	Yes
205	1	0.1695	gi|24113957|ref|NP_7	NP_708467.1	SsrA-binding protein	Yes

206	1	0.1977	gi|24113978|ref|NP_7	NP_708488.1	ribonucleotide reductase stimulatory protein	Yes
207	1	0.2819	gi|56480159|ref|NP_7	NP_708489.2	ribonucleotide-diphosphate reductase subunit alpha	Yes
208	1	0.1678	gi|24113980|ref|NP_7	NP_708490.1	ribonucleotide-diphosphate reductase subunit beta	Yes
209	1	0.2026	gi|24113994|ref|NP_7	NP_708504.1	carbon storage regulator	Yes
210	1	0.4278	gi|56480163|ref|NP_7	NP_708505.2	alanyl-tRNA synthetase	No
211	1	0.3625	gi|24114041|ref|NP_7	NP_708551.1	2-C-methyl-D-erythritol 2,4-cyclodiphosphate synthase	Yes
212	1	0.3373	gi|24114042|ref|NP_7	NP_708552.1	2-C-methyl-D-erythritol 4-phosphate cytidylyltransferase	Yes
213	1	0.2327	gi|24114043|ref|NP_7	NP_708553.1	cell division protein FtsB]	Yes
214	1	0.4374	gi|24114065|ref|NP_7	NP_001361452.1	beta-enolase isoform 1 [Homo sapiens]	No
215	1	0.3964	gi|24114066|ref|NP_7	NP_001896.2	CTP synthase 1 isoform a [Homo sapiens]	No
216	1	0.3224	gi|56480186|ref|NP_7	NP_708604.2	cysteine sulfinate desulfinase	Yes
217	1	0.3236	gi|24114106|ref|NP_7	NP_708616.1	thymidylate synthase	Yes
218	1	0.3624	gi|24114107|ref|NP_7	NP_708617.1	transferase	Yes
219	1	0.427	gi|24114145|ref|NP_7	NP_708655.1	lysyl-tRNA synthetase	No
220	1	0.1868	gi|24114152|ref|NP_7	NP_708662.1	global regulator	Yes
221	1	0.1706	gi|24114165|ref|NP_7	NP_708675.1	D-3-phosphoglycerate dehydrogenase	Yes
222	1	0.2462	gi|56480200|ref|NP_7	NP_708676.2	ribose-5-phosphate isomerase [Homo sapiens]	No
223	1	0.1728	gi|56480206|ref|NP_7	NP_708685.2	fructose-bisphosphate aldolase	Yes
224	1	0.4288	gi|56480207|ref|NP_7	NP_708686.2	phosphoglycerate kinase 1 [Homo sapiens]	No
225	1	0.2434	gi|56480212|ref|NP_7	NP_708699.2	transketolase	Yes
226	1	0.4972	gi|56480215|ref|NP_7	NP_708707.2	S-adenosylmethionine synthase isoform type-2 [Homo sapiens]	No
227	1	0.3372	gi|24114204|ref|NP_7	NP_708714.1	Holliday junction resolvase-like protein	Yes
228	1	0.3617	gi|24114323|ref|NP_7	NP_708833.1	1-acyl-sn-glycerol-3-phosphate acyltransferase	Yes
229	1	0.2164	gi|24114324|ref|NP_7	NP_708834.1	DNA topoisomerase IV subunit A	Yes
230	1	0.3146	gi|56480243|ref|NP_7	NP_708841.2	DNA topoisomerase IV subunit B	Yes
231	1	0.1713	gi|24114342|ref|NP_7	NP_708852.1	3,4-dihydroxy-2-butanone-4-phosphate synthase	Yes
232	1	0.214	gi|24114352|ref|NP_7	XP_006722350.1	ethanolamine-phosphate cytidylyltransferase isoform X1 [Homo sapiens]	No
233	1	0.2739	gi|24114356|ref|NP_7	NP_708866.1	multifunctional tRNA nucleotidyl transferase/2'3'-cyclic phosphodiesterase/2'nucleotidase/phosphatase	Yes
234	1	0.2719	gi|56480250|ref|NP_7	NP_708868.2	bifunctional dihydroneopterin aldolase/dihydroneopterin triphosphate 2'-epimerase	Yes
235	1	0.3093	gi|24114359|ref|NP_7	NP_708869.1	glycerol-3-phosphate acyltransferase PlsY	Yes
236	1	0.4489	gi|24114364|ref|NP_7	NP_708874.1	UGMP family protein	Yes
237	1	0.2439	gi|24114365|ref|NP_7	NP_708875.1	30S ribosomal protein S21	Yes
238	1	0.3936	gi|24114366|ref|NP_7	NP_708876.1	DNA primase	Yes
239	1	0.4689	gi|24114367|ref|NP_7	NP_708877.1	RNA polymerase sigma factor RpoD	Yes
240	1	0.1569	gi|24114437|ref|NP_7	NP_708947.1	SAM-dependent 16S ribosomal RNA C1402 ribose 2'-O-methyltransferase	Yes
241	1	0.3727	gi|24114456|ref|NP_7	NP_708966.1	30S ribosomal protein S15	Yes
242	1	0.2437	gi|56480279|ref|NP_7	NP_708968.2	adenylate cyclase type 3 isoform X15 [Homo sapiens]	yes
243	1	0.4594	gi|24114459|ref|NP_7	NP_001005369.1	translation initiation factor IF-2, mitochondrial isoform a precursor [Homo sapiens]	No
244	1	0.4784	gi|24114460|ref|NP_7	NP_708970.1	transcription elongation factor NusA	Yes
245	1	0.1576	gi|344915328|ref|NP_	NP_708971.4	hypothetical protein SF3211	Yes
246	1	0.3829	gi|24114465|ref|NP_7	NP_708975.1	phosphoglucosamine mutase	Yes
247	1	0.2861	gi|344915329|ref|NP_	NP_708976.4	7,8-dihydropteroate synthase	Yes
248	1	0.4175	gi|24114467|ref|NP_7	NP_708977.1	AFG3-like protein 2 [Homo sapiens]	no

249	1	0.1551	gi|24114468|ref|NP_7	NP_708978.1	rRNA methyltransferase 2, mitochondrial [Homo sapiens]	no
250	1	0.1976	gi|24114470|ref|NP_7	NP_708980.1	transcription elongation factor GreA	Yes
251	1	0.4577	gi|24114472|ref|NP_7	NP_708982.1	mitochondrial ribosome-associated GTPase 2 [Homo sapiens]	no
252	1	0.4484	gi|24114474|ref|NP_7	NP_708984.1	50S ribosomal protein L27	Yes
253	1	0.4063	gi|24114475|ref|NP_7	NP_708985.1	50S ribosomal protein L21	YEs
254	1	0.3626	gi|24114476|ref|NP_7	NP_708986.1	octaprenyl diphosphate synthase	Yes
255	1	0.3843	gi|24114478|ref|NP_7	NP_708988.1	UDP-N-acetylglucosamine 1-carboxyvinyltransferase	Yes
256	1	0.2308	gi|56480284|ref|NP_7	NP_708999.2	lipopolysaccharide transport periplasmic protein LptA	Yes
257	1	0.1864	gi|24114490|ref|NP_7	NP_709000.1	ABC transporter ATP-binding protein	Yes
258	1	0.4077	gi|24114517|ref|NP_7	NP_709027.1	mitochondrial isoform X1 [Homo sapiens]	no
259	1	0.4891	gi|56480292|ref|NP_7	NP_709028.2	39S ribosomal protein L13, mitochondrial [Homo sapiens]	no
260	1	0.1728	gi|24114522|ref|NP_7	NP_709032.1	serine endoprotease	Yes
261	1	0.2339	gi|24114534|ref|NP_7	NP_709044.1	rod shape-determining protein MreD	Yes
262	1	0.2317	gi|24114535|ref|NP_7	NP_709045.1	myc box-dependent-interacting protein 1 isoform 8 [Homo sapiens]	no
263	1	0.26	gi|344915333|ref|NP_		rod shape-determining protein MreB [Shigella flexneri]	no
264	1	0.3848	gi|24114540|ref|NP_7	NP_709050.1	propionyl-CoA carboxylase alpha chain, mitochondrial isoform e precursor [Homo sapiens]	no
265	1	0.4084	gi|56480299|ref|NP_7	NP_709051.2	methylcrotonoyl-CoA carboxylase subunit alpha, mitochondrial isoform X1 [Homo sapiens]	no
266	1	0.3848	gi|56480306|ref|NP_7	NP_709070.2	tRNA(ANN) t(6)A37 threonylcarbamoyladenosine modification protein	Yes
267	1	0.4269	gi|24114564|ref|NP_7	NP_709074.1	peptide deformylase	Yes
268	1	0.4281	gi|24114565|ref|NP_7	NP_709075.1	methionyl-tRNA formyltransferase	Yes
269	1	0.4699	gi|24114572|ref|NP_7	NP_709082.1	50S ribosomal protein L17	Yes
270	1	0.4796	gi|24114573|ref|NP_7	NP_709083.1	DNA-directed RNA polymerase subunit alpha	Yes
271	1	0.4496	gi|56480311|ref|NP_7	NP_709084.2	30S ribosomal protein S4	Yes
272	1	0.4988	gi|24114575|ref|NP_7	NP_709085.1	mitochondrial isoform d [Homo sapiens]	no
273	1	0.4295	gi|24114576|ref|NP_7	NP_709086.1	30S ribosomal protein S13	Yes
274	1	0.3829	gi|24114577|ref|NP_7	NP_709087.1	mitochondrial isoform X1 [Homo sapiens]	no
275	1	0.4784	gi|24114578|ref|NP_7	NP_709088.1	preprotein translocase subunit SecY	Yes
276	1	0.4801	gi|24114579|ref|NP_7	NP_709089.1	50S ribosomal protein L15	Yes
277	1	0.4196	gi|24114580|ref|NP_7	NP_709090.1	50S ribosomal protein L30	Yes
278	1	0.4984	gi|24114581|ref|NP_7	NP_709091.1	30S ribosomal protein S5	Yes
279	1	0.47	gi|24114582|ref|NP_7	NP_709092.1	50S ribosomal protein L18	Yes
280	1	0.4988	gi|24114583|ref|NP_7	NP_709093.1	MULTISPECIES: 50S ribosomal protein L6 [Proteobacteria]	no
281	1	0.4496	gi|24114584|ref|NP_7	NP_709094.1	30S ribosomal protein S8	Yes
282	1	0.4081	gi|24114585|ref|NP_7	NP_709095.1	28S ribosomal protein S14, mitochondrial [Homo sapiens]	No
283	1	0.5169	gi|24114586|ref|NP_7	NP_709096.1	50S ribosomal protein L5	Yes
284	1	0.4699	gi|24114587|ref|NP_7	NP_709097.1	50S ribosomal protein L24	Yes
285	1	0.4891	gi|24114588|ref|NP_7	NP_709098.1	50S ribosomal protein L14	Yes
286	1	0.47	gi|24114589|ref|NP_7	NP_709099.1	40S ribosomal protein S11 [Homo sapiens]	No
287	1	0.4699	gi|24114590|ref|NP_7	NP_709100.1	50S ribosomal protein L29	Yes
288	1	0.4988	gi|24114591|ref|NP_7	NP_709101.1	50S ribosomal protein L16	Yes
289	1	0.4891	gi|56480312|ref|NP_7	NP_709102.2	30S ribosomal protein S3	Yes
290	1	0.4891	gi|24114593|ref|NP_7	NP_709103.1	50S ribosomal protein L22	Yes
291	1	0.47	gi|24114594|ref|NP_7	NP_709104.1	30S ribosomal protein S19	Yes
292	1	0.4796	gi|24114595|ref|NP_7	NP_709105.1	mitochondrial isoform X1 [Homo sapiens]	No
293	1	0.4496	gi|24114596|ref|NP_7	NP_709106.1	50S ribosomal protein L23	Yes
294	1	0.4784	gi|24114597|ref|NP_7	NP_709107.1	50S ribosomal protein L4	Yes

295	1	0.4589	gi|24114598|ref|NP_7	NP_709108.1	50S ribosomal protein L3	Yes
296	1	0.4488	gi|24114599|ref|NP_7	NP_709109.1	30S ribosomal protein S10	Yes
297	1	0.4785	gi|344915334|ref|NP_	NP_709114.2	elongation factor G, mitochondrial isoform 2 [Homo sapiens]	No
298	1	0.5169	gi|24114605|ref|NP_7	NP_709115.1	30S ribosomal protein S7	Yes
299	1	0.4695	gi|56480314|ref|NP_7	NP_709116.2	mitochondrial precursor [Homo sapiens]	No
300	1	0.1576	gi|24114608|ref|NP_7	NP_709118.1	sulfur relay protein TusC	No
301	1	0.4377	gi|24114647|ref|NP_7	NP_709157.1	mitochondrial isoform 3 [Homo sapiens]	No
302	1	0.2299	gi|24114649|ref|NP_7	NP_709159.1	ribulose-phosphate 3-epimerase isoform 1 [Homo sapiens]	No
303	1	0.23	gi|344915336|ref|NP_	NP_709163.4	shikimate kinase I	YEs
304	1	0.2328	gi|56480340|ref|NP_7	NP_709209.2	aspartate-semialdehyde dehydrogenase	Yes
305	1	0.26	gi|24114721|ref|NP_7	NP_709231.1	RNA polymerase factor sigma-32	Yes
306	1	0.2592	gi|56480348|ref|NP_7	NP_709232.2	cell division ABC transporter subunit FtsX	Yes
307	1	0.2594	gi|56480349|ref|NP_7	NP_709233.2	ATP-binding cassette sub-family B member 6, mitochondrial isoform 2 [Homo sapiens]	no
308	1	0.4984	gi|24114724|ref|NP_7	NP_709234.1	signal recognition particle-docking protein FtsY	Yes
309	1	0.1563	gi|24114730|ref|NP_7	NP_709240.1	sulfur transfer protein SirA	Yes
310	1	0.338	gi|24114835|ref|NP_7	NP_709345.1	glycyl-tRNA synthetase subunit beta	Yes
311	1	0.3727	gi|24114836|ref|NP_7	NP_709346.1	glycyl-tRNA synthetase subunit alpha	Yes
312	1	0.2743	gi|24114877|ref|NP_7	NP_709387.1	NAD(P)H-dependent glycerol-3-phosphate dehydrogenase	Yes
313	1	0.229	gi|56480386|ref|NP_7	NP_709391.2	2,3-bisphosphoglycerate-independent phosphoglycerate mutase	Yes
314	1	0.3518	gi|24114902|ref|NP_7	NP_709412.1	DNA-(apurinic or apyrimidinic site) lyase [Homo sapiens]	No
315	1	0.3862	gi|24114903|ref|NP_7	NP_709413.1	phosphopantetheine adenylyltransferase	Yes
316	1	0.4069	gi|24114906|ref|NP_7	NP_709416.1	50S ribosomal protein L28	Yes
317	1	0.4183	gi|344915350|ref|NP_	NP_709418.3	flavoprotein	Yes
318	1	0.2858	gi|24114909|ref|NP_7	NP_709419.1	deoxyuridine 5'-triphosphate nucleotidohydrolase, mitochondrial isoform 3 [Homo sapiens]	No
319	1	0.4277	gi|56480395|ref|NP_7	NP_709428.2	guanylate kinase isoform b [Homo sapiens]	No
320	1	0.2432	gi|24114920|ref|NP_7	NP_709430.1	guanosine-3',5'-bis(diphosphate) 3'-pyrophosphohydrolase MESH1 isoform 2 [Homo sapiens]	No
321	1	0.4394	gi|56480409|ref|NP_7	NP_709495.2	insertase	Yes
322	1	0.4061	gi|24114986|ref|NP_7	NP_709496.1	ribonuclease P	Yes
323	1	0.4286	gi|24114987|ref|NP_7	NP_709497.1	50S ribosomal protein L34	Yes
324	1	0.4497	gi|24114988|ref|NP_7	NP_709498.1	chromosome replication initiator DnaA	Yes
325	1	0.3842	gi|56480410|ref|NP_7	NP_709499.2	DNA polymerase III subunit beta	Yes
326	1	0.4984	gi|56480411|ref|NP_7	NP_709501.2	DNA gyrase subunit B	Yes
327	1	0.3476	gi|24115032|ref|NP_7	NP_709542.1	glutamine--fructose-6-phosphate aminotransferase [isomerizing] 1 isoform 2 [Homo sapiens]	no
328	1	0.4065	gi|24115033|ref|NP_7	NP_709543.1	phosphate uridyltransferase/glucosamine-1-phosphate acetyltransferase	Yes
329	1	0.2291	gi|56480423|ref|NP_7	NP_709544.2	ATP synthase F0F1 subunit epsilon	Yes
330	1	0.3095	gi|24115035|ref|NP_7	NP_709545.1	mitochondrial precursor [Homo sapiens]	No
331	1	0.3095	gi|24115036|ref|NP_7	NP_709546.1	ATP synthase F0F1 subunit gamma	Yes
332	1	0.3102	gi|24115037|ref|NP_7	NP_709547.1	ATP synthase subunit alpha, mitochondrial isoform a precursor [Homo sapiens]	No
333	1	0.17	gi|24115038|ref|NP_7	NP_709548.1	ATP synthase F0F1 subunit delta	Yes
334	1	0.3094	gi|24115039|ref|NP_7	NP_709549.1	ATP synthase F0F1 subunit B	Yes
335	1	0.1711	gi|24115040|ref|NP_7	NP_709550.1	ATP synthase F0F1 subunit C	Yes
336	1	0.2291	gi|24115041|ref|NP_7	NP_709551.1	ATP synthase F0F1 subunit A	Yes
337	1	0.1678	gi|24115044|ref|NP_7	NP_709554.1	protein MTO1 homolog, mitochondrial isoform a [Homo sapiens]	No

338	1	0.1718	gi|56480432|ref|NP_7	NP_709581.2	ATP-dependent DNA helicase Rep	Yes
339	1	0.2426	gi|56480434|ref|NP_7	NP_709584.2	thioredoxin, mitochondrial precursor [Homo sapiens]	No
340	1	0.2152	gi|56480435|ref|NP_7	NP_709606.1	uroporphyrinogen-III synthase	Yes
341	1	0.2606	gi|24115096|ref|NP_7	NP_709607.2	porphobilinogen deaminase	Yes
342	1	0.3111	gi|56480439|ref|NP_7	NP_709616.3	diaminopimelate epimerase	Yes
343	1	0.2156	gi|344915361|ref|NP_	NP_709616.3	diaminopimelate epimerase	Yes
344	1	0.1998	gi|56480441|ref|NP_7	NP_709620.2	DNA-dependent helicase II	Yes
345	1	0.322	gi|24115129|ref|NP_7	NP_709639.1	2-methoxy-6-polyprenyl-1,4-benzoquinol methylase, mitochondrial precursor [Homo sapiens]	No
346	1	0.2032	gi|56480451|ref|NP_7	NP_709640.2	hypothetical protein SF3912	Yes
347	1	0.2019	gi|24115131|ref|NP_7	NP_709641.1	ubiquinone biosynthesis protein UbiB	Yes
348	1	0.1704	gi|24115134|ref|NP_7	NP_709644.1	twin-arginine protein translocation system subunit TatC	Yes
349	1	0.1869	gi|24115137|ref|NP_7	NP_709647.1	3-octaprenyl-4-hydroxybenzoate decarboxylase	Yes
350	1	0.1728	gi|24115144|ref|NP_7	NP_709654.1	protoporphyrinogen oxidase	Yes
351	1	0.2831	gi|24115152|ref|NP_7	NP_709662.1	DNA polymerase I	Yes
352	1	0.4699	gi|344915363|ref|NP_	NP_709663.4	large subunit GTPase 1 homolog isoform X1 [Homo sapiens]	No
353	1	0.2446	gi|24115158|ref|NP_7	NP_709668.1	glutamine synthetase	Yes
354	1	0.2563	gi|56480481|ref|NP_7	NP_709721.2	phosphofructokinase, platelet type isoform 4 [Homo sapiens]	No
355	1	0.3587	gi|24115214|ref|NP_7	NP_709724.1	triosephosphate isomerase isoform 1 [Homo sapiens]	No
356	1	0.1845	gi|56480486|ref|NP_7	NP_709739.2	primosome assembly protein PriA	Yes
357	1	0.3092	gi|24115230|ref|NP_7	NP_709740.1	50S ribosomal protein L31	Yes
358	1	0.2327	gi|344915367|ref|NP_	NP_709771.4	glutamate racemase	Yes
359	1	0.3849	gi|24115262|ref|NP_7	NP_709772.1	acetylenolpyruvoylglucosamine reductase	Yes
360	1	0.3504	gi|24115263|ref|NP_7	NP_709773.1	biotin--protein ligase isoform X2 [Homo sapiens]	Yes
361	1	0.155	gi|56480499|ref|NP_7	NP_709774.2	pantothenate kinase	Yes
362	1	0.2548	gi|24115265|ref|NP_7	NP_709775.1	elongation factor Tu, mitochondrial isoform 1 precursor [Homo sapiens]	No
363	1	0.314	gi|24115266|ref|NP_7	NP_709776.1	preprotein translocase subunit SecE	Yes
364	1	0.3624	gi|24115267|ref|NP_7	NP_709777.1	NusG	Yes
365	1	0.4171	gi|24115268|ref|NP_7	NP_709778.1	mitochondrial isoform a [Homo sapiens]	No
366	1	0.3102	gi|24115269|ref|NP_7	NP_709779.1	50S ribosomal protein L1	Yes
367	1	0.4277	gi|24115270|ref|NP_7	NP_709780.1	50S ribosomal protein L10	Yes
368	1	0.4789	gi|24115271|ref|NP_7	NP_709781.1	50S ribosomal protein L7/L12	Yes
369	1	0.4988	gi|56480500|ref|NP_7	NP_709782.2	DNA-directed RNA polymerase subunit beta	Yes
370	1	0.4785	gi|24115273|ref|NP_7	NP_709783.1	DNA-directed RNA polymerase subunit beta'	Yes
371	1	0.2591	gi|24115281|ref|NP_7	NP_709791.1	uroporphyrinogen decarboxylase [Homo sapiens]	No
372	1	0.4484	gi|56480523|ref|NP_7	NP_709860.2	single-stranded DNA-binding protein	Yes
373	1	0.4695	gi|24115358|ref|NP_7	NP_709868.1	replicative DNA helicase	Yes
374	1	0.2028	gi|24115367|ref|NP_7	NP_709877.1	LexA repressor	Yes
375	1	0.2168	gi|344915376|ref|NP_	NP_709879.4	glycerol-3-phosphate acyltransferase	Yes
376	1	0.2584	gi|24115370|ref|NP_7	NP_709880.1	octaprenyltransferase	Yes
377	1	0.2817	gi|56480535|ref|NP_7	NP_709894.2	glucose-6-phosphate isomerase isoform 3 [Homo sapiens]	No
378	1	0.3146	gi|56480550|ref|NP_7	NP_709939.2	lipopolysaccharide ABC transporter permease	Yes
379	1	0.2612	gi|56480551|ref|NP_7	NP_709940.2	lipopolysaccharide ABC transporter permease LptF	Yes
380	1	0.4891	gi|24115433|ref|NP_7	NP_709943.1	valine--tRNA ligase isoform X1 [Homo sapiens]	No
381	1	0.1854	gi|24115435|ref|NP_7	NP_709945.1	N-acetylaspartate synthetase [Homo sapiens]	No
382	1	0.1699	gi|24115458|ref|NP_7	NP_709968.1	ryanodine receptor 3 isoform X12 [Homo sapiens]	No

383	1	0.1709	gi|56480562|ref|NP_7	NP_709969.2	fructose-1,6-bisphosphatase 1 [Homo sapiens]	No
384	1	0.3385	gi|56480564|ref|NP_7	NP_709975.2	inorganic pyrophosphatase	Yes
385	1	0.4283	gi|24115497|ref|NP_7	NP_710007.1	10 kDa heat shock protein, mitochondrial [Homo sapiens]	No
386	1	0.3339	gi|24115498|ref|NP_7	NP_710008.1	60 kDa heat shock protein, mitochondrial [Homo sapiens]	No
387	1	0.227	gi|24115504|ref|NP_7	NP_710014.1	elongation factor P	Yes
388	1	0.2589	gi|24115518|ref|NP_7	NP_710028.1	phosphatidylserine decarboxylase	Yes
389	1	0.2867	gi|344915382|ref|NP_	NP_710030.4	oligoribonuclease, mitochondrial precursor [Homo sapiens]	No
390	1	0.4177	gi|24115523|ref|NP_7	NP_710033.1	ADP-binding protein	Yes
391	1	0.2158	gi|24115526|ref|NP_7	NP_710036.1	tRNA delta(2)-isopentenylpyrophosphate transferase	Yes
392	1	0.3948	gi|24115554|ref|NP_7	NP_710036.1	30S ribosomal protein S6	Yes
393	1	0.4291	gi|24115555|ref|NP_7	NP_710065.1	30S ribosomal protein S18	Yes
394	1	0.1694	gi|24115556|ref|NP_7	NP_710066.1	50S ribosomal protein L9	Yes
395	1	0.1869	gi|24115590|ref|NP_7	NP_710100.1	DNA replication protein DnaC	Yes
